# Ureteral Embolization to Prevent Thrombotic Obstruction of Nephrostomy Catheters in the Context of Refractory Hemorrhagic Radiation Cystitis with Severe Vesicoureteral Reflux

**DOI:** 10.1155/2016/2186049

**Published:** 2016-02-25

**Authors:** Vidhush Yarlagadda, Keith Pettibon, Nathan Ertel, Jeffrey Nix

**Affiliations:** University of Alabama Birmingham Medical Center, 510 20th Street South, Birmingham, AL 35233, USA

## Abstract

We present a case of refractory hemorrhagic radiation cystitis in a patient who failed conservative management and was unable to undergo operative urinary diversion secondary to multiple comorbidities. His management was complicated by recurrent obstruction of his nephrostomy catheters due to marked ureteral thrombus formation from blood refluxing into the ureters from the urinary bladder. We were successful in treating his condition by occluding his distal ureters with a combination of embolization coils and glue to prevent the reflux of blood in order to allow his nephrostomy catheters to function properly.

## 1. Introduction

Hemorrhagic radiation cystitis is a difficult problem for urologists to treat. The etiology is variable; however the common factor involved in most cases is a diffuse inflammatory response of the bladder mucosa. The difficulty in treatment is in part due to the refractory nature of the disease to conservative management [1]. Standard conservative measures include hyperbaric oxygen treatment and intravesical instillation with various agents including aminocaproic acid, aluminum salts, and Formalin. For patients with refractory disease severe enough to cause urinary retention secondary to clot obstruction, it is imperative to divert urinary flow to protect renal function. This can be done with percutaneous nephrostomy catheter placement or more definitively with cystectomy and ileal conduit [2]. In patients with refractory disease that are nephrostomy catheter dependent, clot obstruction can present a difficult complication. Here we describe a novel approach to this vexing problem.

## 2. Case Report

**Figure 1:**
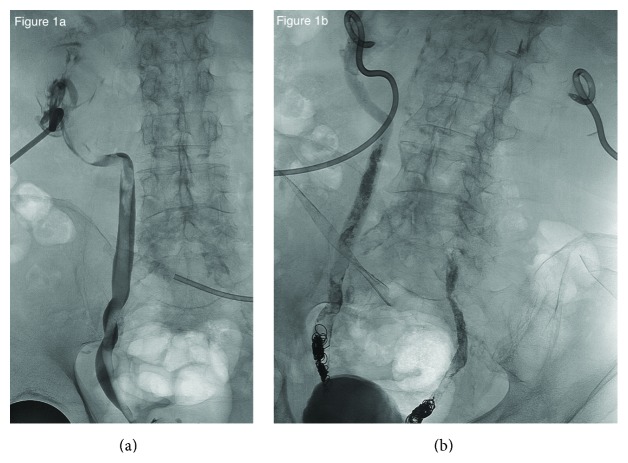
(a) Nephroureterogram through the indwelling left nephrostomy catheter shows moderate dilation of the left ureter with multiple large, irregular filling defects throughout the collecting system and ureter representing the thrombus burden. The contracted urinary bladder containing a large thrombus burden is also shown. (b) Postembolization fluoroscopic image showing occlusion of both distal ureters with a combination of coils and glue.

A 78-year-old man with ischemic congestive heart failure (CHF) and chronic obstructive pulmonary disease (COPD) presented to our institution with refractory hemorrhagic cystitis that developed after external beam radiation therapy (EBRT) for Gleason 3+4 prostate cancer. Prior to presentation at our hospital, he had undergone multiple cystoscopic clot evacuations, fulguration, and failed therapy with systemic and intravesical Amicar. During his hospitalization, he experienced significant hemorrhage requiring multiple blood transfusions and pressor support. Bilateral nephrostomy catheters were placed due to recurrent episodes of clot retention despite continuous bladder irrigation. The nephrostomy catheters became obstructed with clot almost immediately. Despite being exchanged for larger catheters and flushed hourly, the catheters continued to become obstructed. The patient was unable to undergo urinary diversion due to his age and comorbidities and therefore underwent 1% Formalin instillation of the urinary bladder. During this procedure it was noted that both of the ureteral orifices were extremely large and easily refluxed, so much so that a 36 French balloon dilator was required for ureteral obstruction during instillation. He was discharged home with apparent resolution of hemorrhage but was subsequently readmitted for a hip fracture following a fall likely caused by hemorrhagic anemia from recurrent hemorrhage. He underwent repeat intravesical instillation with 2% Formalin, and, again, it was noted that he had massively dilated ureteral orifices which were difficult to obstruct. He continued to have persistent bleeding and obstruction of his nephrostomy catheters, which was thought to be due to reflux of blood from the bladder into the collecting system due to his dilated ureteral orifices. Given the refractory and persistent nature of hemorrhage from the collecting system and exhaustion of all other viable options, the decision was made to attempt bilateral embolization of the ureters. Using the existing access to the renal collecting system from the nephrostomy catheters, the distal ureters were successfully embolized using a combination of 0.035 and 0.038 pushable coils followed by NBCA (N-*butyl*-cyanoacrylate) glue (Figure 1). The collecting systems were flushed repeatedly to remove as much of the preexisting clot burden as possible. Postprocedure bilateral nephrostograms demonstrated complete occlusion of the ureters with no passage of contrast into the bladder. The following day, there was still minimal output from his nephrostomy catheters, and his creatinine remained mildly elevated. Follow-up nephrostograms demonstrated residual clot within both collecting systems, with continued occlusion of the distal ureters by the coils and glue. tPA was instilled into both collecting systems to address the residual clot burden. Following the procedure, there was improved output from both nephrostomy catheters, and the patient's creatinine began trending downward appropriately. He was seen in the clinic 3 weeks later. His nephrostomy catheters were flushing without difficulty and putting out relatively equal amounts of clear urine. His laboratory studies at that time indicated intact renal function with resolution of postrenal acute kidney injury (AKI).

## 3. Discussion

We have described a case of refractory hemorrhagic cystitis complicated by ureteral reflux in a patient that was unfit to undergo urinary diversion and failed less invasive therapy. This reflux prevented nephrostomy catheter function due to recurrent clotting within the catheters as well as the renal collecting system. In this patient with significant comorbidities and short life expectancy, our goal was to prevent nephrostomy catheter obstruction in order to preserve renal function. Ureteral embolization is a known intervention in patients with ureteroarterial and ureterovaginal fistulas as well as those with persistent ileoureteral leaks [3–5]. However, there have been no reports of its use in cases of refractory bladder hemorrhage with reflux into the collecting system preventing nephrostomy catheter drainage. Prior reports of the durability of coils have been documented in the interventional radiology literature [6]. Based on our experience, ureteral embolization can be utilized in cases such as this when all other options have been exhausted and definitive operative intervention is not feasible.
